# Accuracy of Physician Estimates of Out-of-Pocket Costs for Medication Filling

**DOI:** 10.1001/jamanetworkopen.2021.33188

**Published:** 2021-11-05

**Authors:** Caroline E. Sloan, Lorena Millo, Sophia Gutterman, Peter A. Ubel

**Affiliations:** 1 Department of Medicine, Duke University School of Medicine, Durham, North Carolina; 2 Health Services Research and Development Center of Innovation, Durham Veterans Affairs Health Care System, Durham, North Carolina; 3 School of Medicine, University of North Carolina at Chapel Hill, Chapel Hill; 4 School of Medicine, University of Michigan, Ann Arbor; 5 Fuqua School of Business, Duke University, Durham, North Carolina; 6 Sanford School of Public Policy, Duke University, Durham, North Carolina

## Abstract

**Importance:**

One-third of US residents have trouble paying their medical bills. They often turn to their physicians for help navigating health costs and insurance coverage.

**Objective:**

To determine whether physicians can accurately estimate out-of-pocket expenses when they are given all of the necessary information about a drug’s price and a patient’s insurance plan.

**Design, Setting, and Participants:**

This national mail-in survey used a random sample of US physicians. The survey was sent to 900 outpatient physicians (300 each of primary care, gastroenterology, and rheumatology). Physicians were excluded if they were in training, worked primarily for the Veterans Administration or Indian Health Service, were retired, or reported 0% outpatient clinical effort. Analyses were performed from July to December 2020.

**Main Outcomes and Measures:**

In a hypothetical vignette, a patient was prescribed a new drug costing $1000/month without insurance. A summary of her private insurance information was provided, including the plan’s deductible, coinsurance rates, copays, and out-of-pocket maximum. Physicians were asked to estimate the drug’s out-of-pocket cost at 4 time points between January and December, using the plan’s 4 types of cost-sharing: (1) deductibles, (2) coinsurance, (3) copays, and (4) out-of-pocket maximums. Multivariate linear regression was used to assess differences in performance by specialty, adjusting for attitudes toward cost conversations, demographics, and clinical characteristics.

**Results:**

The response rate was 45% (405 of 900) and 371 respondents met inclusion criteria. Among the respondents included in this study, 59% (n = 220) identified as male, 23% (n = 84) as Asian, 3% (n = 12) as Black, 6% (n = 24) as Hispanic, and 58% (n = 216) as White; 30% (n = 112) were primary care physicians, 35% (n = 128) were gastroenterologists, and 35% (n = 131) were rheumatologists; and the mean (SD) age was 49 (10) years. Overall, 52% of physicians (n = 192) accurately estimated costs before the deductible was met, 62% (n = 228) accurately used coinsurance information, 61% (n = 224) accurately used copay information, and 57% (n = 210) accurately estimated costs once the out-of-pocket maximum was met. Only 21% (n = 78) of physicians answered all 4 questions correctly. Ability to estimate out-of-pocket costs was not associated with specialty, attitudes toward cost conversations, or clinic characteristics.

**Conclusions and Relevance:**

This survey study found that many US physicians have difficulty estimating out-of-pocket costs, even when they have access to their patients' insurance plans. The mechanics involved in calculating real-time out-of-pocket costs are complex. These findings suggest that increased price transparency and simpler insurance cost-sharing mechanisms are needed to enable informed cost conversations at the point of prescribing.

## Key Points

**Question:**

Can physicians accurately estimate a patient’s out-of-pocket expenses if they are given all the necessary information about a drug’s price and the patient’s insurance plan?

**Findings:**

In this survey study of 371 primary care physicians, gastroenterologists, and rheumatologists, only 21% could accurately estimate out-of-pocket drug costs using information about the drug’s price and an insurance plan’s cost-sharing mechanisms, including deductibles, copays, coinsurance, and out-of-pocket maximums.

**Meaning:**

These findings suggest that few physicians are able to estimate out-of-pocket costs accurately enough to have informed conversations about financial trade-offs with their patients.

## Teaser

This survey study examines whether physicians can accurately estimate out-of-pocket expenses when they are given all of the necessary information about a drug’s price and a patient’s insurance plan.

## Introduction

Over the last 2 decades, out-of-pocket expenses have risen dramatically in the US due to a combination of increasing health care prices and increasing insurance cost-sharing requirements.^1^ As a result, one-third of US residents have trouble paying their medical bills, even when they have insurance.^2^ Patients who struggle to afford their medications may cut pills in half, take them every other day, or forego them altogether.^3,4,5^ Cost-related nonadherence is associated with a higher risk of financial instability, disease progression, hospitalization, and death.^6,7,8,9^

To make matters worse, out-of-pocket costs are often difficult to predict, leaving patients and clinicians unable to account for costs in their medical decisions.^10,11^ The out-of-pocket cost associated with a single medication might change multiple times between January and December in a given year, depending on the amount of care a patient’s insurance plan has already covered.^12^ For example, early in the year, a patient who has not yet met her prescription drug deductible might pay the full price for her prescription drugs. Later in the year, once she has met her deductible, she likely pays either a flat fee (copay) or a percentage of each drug’s cost (coinsurance). If she incurs enough medical expenses, she may reach her out-of-pocket maximum before the end of the year. Once she meets this limit, she pays nothing at all for prescription drugs for the rest of the year. Such cost-sharing mechanisms exist to address moral hazard and prevent overconsumption of medical resources, but their complexity often confuses patients,^13^ who turn to their clinicians for assistance.^14,15,16,17,18,19,20^ Although patients may not expect clinicians to have all the answers, they do report hoping for changes in prescription patterns or referrals to pharmaceutical assistance programs when out-of-pocket costs are projected to be high.^21,22^

Physician experts and patient advocacy groups have recommended that communication about the financial trade-offs of potential treatment options become part of routine clinical practice.^23,24,25,26,27^ Cost conversations have the potential to improve relationships between patients and physicians, decrease out-of-pocket costs, and improve outcomes, especially when those costs are known ahead of time.^28,29^ Although prior work has shown that physicians agree that discussing costs is important,^22^ it is still unclear whether physicians understand insurance cost-sharing mechanisms well enough to have informed cost conversations with their patients.^30^ In this study, we report the results of a survey aimed at determining whether physicians can accurately estimate a patient’s out-of-pocket expenses when they are given all the necessary information about a medication’s price and a patient’s insurance plan.

## Methods

### Study Design

We surveyed 300 primary care physicians (internal medicine or family medicine), 300 gastroenterologists, and 300 rheumatologists who were randomly selected from the American Medical Association (AMA) Masterfile of all licensed physicians in the US. Data were obtained from Redi-Data, an AMA authorized database licensee. The confidential survey was sent by mail to each physician’s home address in December 2019 with a $5 cash incentive. Survey recipients were told that the goal of the survey was “to understand how physicians think about discussing out-of-pocket costs with their patients” and that their answers would be anonymous. A second wave of surveys was sent to nonrespondents in March 2020. We excluded physicians who were in training, worked primarily for the Veterans Administration or Indian Health Service, were retired, or reported 0% outpatient clinical effort.

We entered survey responses into an electronic database, using Qualtrics software. Each survey was entered twice, by 2 different people. Any discrepancies between duplicate entries were reconciled by referencing the original survey.

The Duke University institutional review board approved the study protocol and deemed that voluntary completion of the questionnaire reflected consent to participate in the study. This study followed the American Association for Public Opinion Research (AAPOR) reporting guideline for calculating response rate.

### Survey Development

The survey was extensively pretested with primary care physicians and internal medicine subspecialists to assess its overall length and clarity. The number and wording of the questions were modified based on their verbal and written feedback, as well as a pilot study sent to a random sample of 300 physicians prior to final survey dissemination. This iterative process led to deletion of redundant questions and clarifications about the types of out-of-pocket costs physicians were being asked to estimate (eg, specific medications, not premiums). The full version of the survey that was sent to primary care physicians is shown in the eFigure in the Supplement.

### Demographic and Practice Characteristics

We collected information on demographics (age, year of medical school graduation, gender, race, ethnicity) and practice characteristics (percentage clinical time, payer mix, academic affiliation, access to a social worker, use of an electronic health record [EHR], access to out-of-pocket cost information via the EHR). Respondents could write in other gender and race categories if those included in the survey were not applicable to them.

### Attitudes Toward Cost Conversations

Physicians were given 9 statements assessing their attitudes toward having cost conversations with patients. Response options used a 6-point Likert scale ranging from strongly disagree to strongly agree. These statements addressed their thoughts on the cost conversations themselves (eg, obligation to initiate discussions, ability to lower out-of-pocket costs), barriers to effective cost conversations (eg, insufficient time, insufficient knowledge, discomfort with the topic), and perception of patients’ expectations regarding cost conversations. Statements were adapted from a 2003 study conducted by Alexander and colleagues.^22,31^

### Ability to Estimate Out-of-Pocket Costs

**Figure 1.**
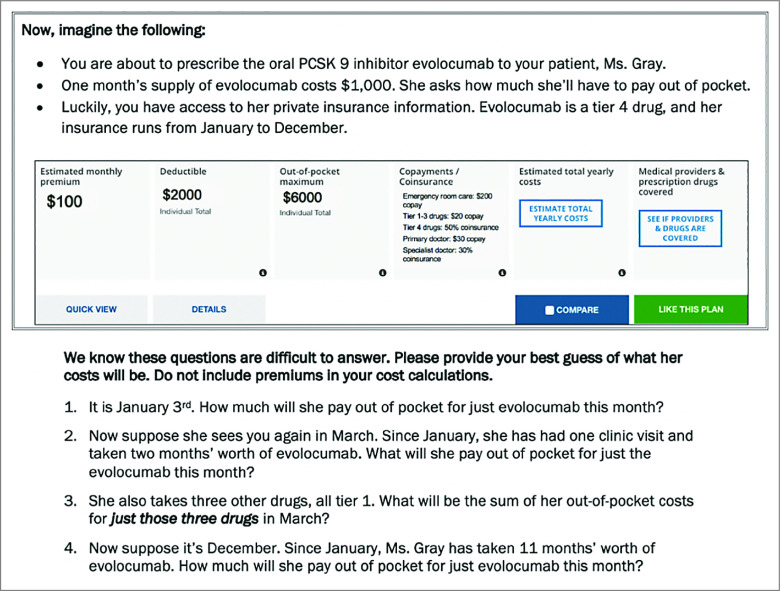
Vignette Assessing Physicians’ Ability to Estimate Out-of-Pocket Costs This figure shows the vignette that primary care physicians received. In the vignette that gastroenterologists received, the medication was adalimumab. In the vignette that rheumatologists received, the medication was tofacitinib. Four questions asked physicians to estimate how much the patient would have to pay out of pocket at 4 time points between January and December. Physicians were told that the patient consistently picked up her medications every month. The aim of the 4 questions tied to the vignette was to capture physicians’ ability to estimate out-of-pocket costs using 4 types of insurance cost-sharing: (1) deductibles, (2) coinsurance, (3) copays, and (4) out-of-pocket maximums. The insurance information is presented in the same format used by insurance plans available on the HealthCare.gov website. The insurance plan ran from January to December and the new drug was listed as tier 4 on the plan’s formulary. The first question asked how much she would pay for the tier 4 drug in January. In January, she would not have met her deductible of $2000, so she would have to pay the full price of the drug: $1000. The second question asked how much she would pay for the drug in March. By this time, she would have met her deductible by paying $1000 in January and $1000 in February, so she would owe the coinsurance for tier 4 drugs (50%): $500. The third question asked how much she would pay for 3 other tier 1 drugs available through an outpatient pharmacy benefit in March. Her insurance plan indicated a $20 copay per tier 1 drug, so she would owe a total of $60 for these 3 drugs. Finally, the fourth question asked how much she would pay for the new drug in December. By then, she would have met her out-of-pocket maximum of $6000, so she would owe nothing for the drug ($0).

Physicians’ ability to estimate out-of-pocket costs was assessed using a short patient vignette (Figure 1). In the vignette, a hypothetical patient was prescribed a new tier 4 drug that cost $1000/month without insurance. Vignettes were tailored to physician specialty: primary care physicians prescribed the lipid-lowering medication evolocumab to a patient with hyperlipidemia, gastroenterologists prescribed the TNF-α inhibitor adalimumab to a patient with inflammatory bowel disease, and rheumatologists prescribed the oral disease-modifying antirheumatic drug tofacitinib to a patient with rheumatoid arthritis. A summary of the patient’s private insurance information was provided, including deductible, copays, coinsurance rates, and out-of-pocket maximum.

Four questions asked physicians to estimate how much the patient would have to pay out of pocket at 4 time points between January and December (see Figure 1 for details). The questions assessed their ability to accurately estimate out-of-pocket costs using 4 types of cost-sharing that Medicare and private insurance plans typically use: (1) deductibles, (2) coinsurance, (3) copays, and (4) out-of-pocket maximums. The questions were deliberately designed to be conditional upon each other, to reflect the realities of cost estimations in the clinic.

### Statistical Analysis

We used simple descriptive statistics to evaluate physicians’ demographics, clinical characteristics, and attitudes toward cost conversations. Attitudes toward cost conversations are reported as binary variables, with 1 corresponding to all agree statements and 0 corresponding to all disagree statements. We used exploratory factor analysis with varimax rotation to determine whether questions addressing physicians’ attitudes toward cost conversations could be combined into composite categories. To create variables representing these composite categories, we transformed responses to each attitude question into binary variables.

We determined the percentage of vignette questions answered correctly overall, by question and by specialty. In the baseline analysis, a correct response was defined as an exact match of the estimated cost as described in Figure 1. Incorrect and “I don’t know” responses were combined, because both of these responses indicated that the physician could not estimate the patient’s out-of-pocket cost.

We considered that some respondents who had accurately estimated the patient’s drug-related out-of-pocket cost may have incorrectly included additional out-of-pocket costs in their responses, such as visit copay or monthly premium. In a sensitivity analysis, responses were defined as correct if they fell within prespecified ranges that included premiums, visit copays, and additional drugs mentioned in the question prompts (eTable 1 in the Supplement).

We used multivariate linear regression to explore whether any physician or clinical practice variables were associated with the ability to accurately estimate out-of-pocket costs, defined as proportion of vignette questions answered correctly. The model included key sociodemographic information (gender, race, ethnicity), years of experience, composite categories of attitudes toward cost conversations, percentage outpatient time, academic affiliation, and access to cost information via the EHR. We did not include information on patient payer mix because of a large number of missing data.

All tests were 2-sided and used *P* < .05 as a measure of statistical significance. All statistical analyses were performed with Stata software version 16.0 (StataCorp) from July to December 2020. A prespecified statistical analysis plan was preregistered with Open Science Framework.^32^

## Results

### Recruitment and Respondent Characteristics

Of the 900 physicians surveyed, 22 were unreachable by mail. Of the remaining 878 physicians, 405 returned completed questionnaires, for an overall response rate of 45% (405 of 900).^33^ After excluding 34 physicians who reported 0% outpatient clinical time, being retired, or working for the Veterans Administration, we included 371 physicians in our analysis. Of these, 220 physicians (59%) identified as male, 84 (23%) as Asian, 12 (3%) as Black, 24 (6%) as Hispanic, 216 (58%) as White; and the mean (SD) age was 49 (10) years. The mean (SD) years of experience was 22 (10). Specialties were fairly equally represented, with 112 primary care physicians (30%), 128 gastroenterologists (35%), and 131 rheumatologists (35%). Physicians spent a mean (SD) of 83% (22%) of their time in the outpatient setting. They estimated that a mean (SD) of 72% (24%) of their patients were enrolled in Medicare or private insurance. 352 physicians used an EHR (95%), but only 92 (25%) had access to any out-of-pocket cost or coverage information through an EHR.

Among all survey respondents, 354 (87%) responded to the first wave of the study and 51 (13%) responded to the second wave, which was sent at the beginning of the COVID pandemic in the US. Surveys were anonymous and deidentified, so we were unable to compare the characteristics of physicians who responded to the survey with those of physicians who did not respond.

### Attitudes Toward Cost Conversations

In exploratory factor analysis, we reduced questions on attitudes toward cost conversations to 3 composite categories, labeled as (1) duty, (2) perceived barriers, and (3) perceived expectations (see eTable 2 in the Supplement for rotated factor loadings). Higher scores indicated a higher sense of duty, barriers, and expectations regarding cost conversations, respectively. Most physicians agreed that they had an obligation to initiate cost conversations with their patients (n = 276; 74%), but had a hard time advising their patients about out-of-pocket costs (n = 285; 77%) (Table 1). Barriers included insufficient time (n = 283; 76%), insufficient knowledge (n = 255; 69%), and discomfort with cost conversations (n = 153; 41%). Approximately two-thirds thought their patients expected them to solve their cost-related issues (n = 232; 63%).

**Table 1.** Physician Attitudes Toward Cost Conversations

Physician attitude	Agree, No. (%)^a^
Duty	
Doctors have an obligation to initiate discussions about out-of-pocket costs when writing orders	276 (74)
There is nothing I can do to lower patients’ out-of-pocket costs^b^	124 (33)
It is not my job to deal with patients’ out-of-pocket costs^b^	126 (34)
Perceived barriers	
I have a hard time advising my patients on their out-of-pocket costs	285 (77)
I do not have enough time to discuss patients’ out-of-pocket costs	283 (76)
I usually don’t know how to answer questions about out-of-pocket costs	255 (69)
I know approximately how much my patients are spending on their medical care^b^	99 (27)
I feel uncomfortable discussing out-of-pocket costs with my patients	153 (41)
Perceived expectations	
My patients expect me to solve their cost-related issues during or right after clinic visits	232 (63)

^a^ Sum of “somewhat agree,” “agree,” and “strongly agree.”

^b^ This question was reverse coded in exploratory factor analysis.

### Ability to Estimate Out-of-Pocket Costs

**Figure 2.**
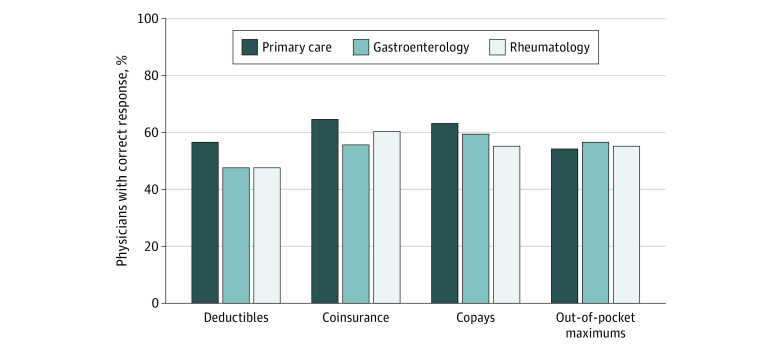
Proportion of Physicians Answering Each Insurance Coverage Question Correctly, by Specialty Overall, 192 physicians (52%) accurately estimated the tier 4 drug’s out-of-pocket cost before the deductible was met; 228 (62%) accurately used coinsurance information to estimate the drug’s out-of-pocket cost once the deductible was met; 224 (61%) accurately used drug copay information to estimate the out-of-pocket costs of 3 other tier 1 drugs; and 210 (57%) accurately estimated the tier 4 drug’s out-of-pocket cost once the out-of-pocket maximum was met.

**Figure 3.**
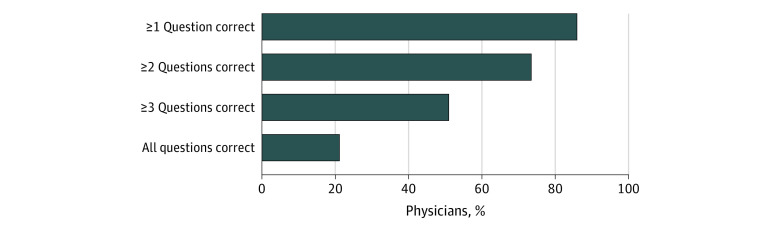
Overall Number of Insurance Coverage Questions Answered Correctly Approximately three-quarters of physicians (74%) answered at least 2 questions correctly, but only 51% (n = 188) answered 3 questions correctly and 21% (n = 78) answered all 4 questions correctly. Three respondents with missing responses to all 4 vignette questions were excluded.

The mean (SD) percentage of cost-sharing questions answered correctly was 58% (33%), indicating that, on average, physicians answered approximately 2 out of the 4 questions correctly. Overall, 192 physicians (52%) accurately estimated the tier 4 drug’s cost before the deductible was met; 228 (62%) accurately used coinsurance information to estimate the drug’s cost once the deductible was met; 224 (61%) accurately used drug copay information to estimate the cost of 3 other tier 1 drugs; and 210 (57%) accurately estimated the tier 4 drug’s cost once the out-of-pocket maximum was met (Figure 2). Only 78 physicians (21%) answered all 4 questions correctly (Figure 3).

In univariate analyses, ability to estimate out-of-pocket costs was not associated with specialty, years of experience, outpatient clinical time, perceived barriers, or perceived expectations. Physicians who felt a duty to engage in cost conversations were more likely to estimate out-of-pocket costs accurately (5.2% [95% CI, 1.7%-8.7%] higher proportion of questions answered correctly; *P* = .003). Access to cost or coverage information in the EHR was associated with improved ability to estimate out-of-pocket costs (11.2% [95% CI, 3.8%-18.5%] higher proportion of questions answered correctly; *P* = .003). After adjustment in multivariate analysis, accurate out-of-pocket cost estimations remained associated with access to cost or coverage information in the EHR (10.1% [95% CI, 2.4%-17.8%] higher proportion of questions answered correctly; *P* = .01) (Table 2).

**Table 2.** Factors Associated With Physician Ability to Estimate Out-of-Pocket Costs in Multivariate Linear Regression

Variable	β coefficient	SE (95% CI)	*P* value
Specialty			
Primary care	[Reference]	[Reference]	[Reference]
Gastroenterology	1.28	4.86 (−8.28 to 10.84)	.79
Rheumatology	−2.68	4.45 (−11.43 to 6.06	.55
Attitudes toward cost conversations			
Duty to discuss costs	3.72	2.08 (−0.38 to 7.82)	.08
Barriers to discussing costs	−0.15	1.28 (−2.67 to 2.38)	.91
Expected to discuss costs	3.25	3.63 (−3.89 to 10.40)	.37
Years of experience	0.00	0.19 (−0.38 to 0.37)	.99
Percent outpatient clinical time	−0.04	0.09 (−0.22 to 0.14)	.66
Academic affiliation	−3.87	4.72 (−13.16 to 5.42)	.41
Use of EHR that provides cost information			
No	[Reference]	[Reference]	[Reference]
Yes	10.10	3.89 (2.45 to 17.76)	.01^a^
Unsure	−10.86	7.32 (−25.26 to 3.53)	.14

Abbreviation: EHR, electronic health record.

^a^ Significant at the α = .05 level.

In the sensitivity analysis that defined responses as correct if they fell within a prespecified range (eTable 1 in the Supplement), mean (SD) percentage of questions answered correctly increased to 62% (33%), and the proportion of respondents considered to have answered all 4 questions correctly increased to 26% (n = 97). Results of multivariate linear regression did not change.

## Discussion

This survey study found that while most US physicians feel it is their duty to discuss costs with their patients, they have considerable difficulty estimating those out-of-pocket costs, even when they are given adequate information about their patients' insurance plans. In particular, they struggle with cost-sharing mechanisms, including deductibles, copays, coinsurance, and out-of-pocket maximums. Their ability to estimate out-of-pocket costs does not vary by specialty.

As drug costs rise and insurance plans increasingly rely on cost-sharing strategies such as deductibles and coinsurance,^34^ patients in the US will likely continue to grapple with trade-offs between management options, or between medical care and nonmedical essentials such as food and housing. One way physicians can help patients make informed decisions is to initiate cost conversations in the clinic.^35^ Theoretically, any physician who has a few minutes to spare can look up a drug’s retail price^36^ and a patient’s insurance benefits, and then give an estimate of that drug’s cost. We found that 69% to 77% of physicians report having a hard time advising their patients on their out-of-pocket costs, answering questions about out-of-pocket costs, and determining how much their patients are spending on medications. More than three-quarters (79%) provided inaccurate estimates for at least one aspect of drug-related cost-sharing obligations. Even though a substantial proportion of physicians responding to our survey agreed that they have a responsibility to engage in cost conversations, this view was not associated with improved ability to estimate drug-related out-of-pocket costs.

Physicians’ difficulties estimating drug-related out-of-pocket costs should not be surprising. After all, health care accounting is not part of the medical school curriculum. Moreover, it can be difficult for physicians to keep up with the ever-changing cost-sharing strategies used by insurance plans. As a result, when cost conversations do occur, they are often unproductive^17,37,38^ or may even be counterproductive if, for example, a physician provides inaccurate information regarding the price of a drug. Future studies should evaluate whether physicians have similar difficulties estimating the costs of other common medical services, including procedures and specialty visits.

Prior studies have shown that accurate out-of-pocket cost information can improve patient decision-making.^29,39,40^ Our findings suggest that health systems should not rely solely on physicians to calculate and discuss costs with patients. Even if physicians did have a better understanding of insurance coverage, they are unlikely to have the spare time to calculate their patients’ expected costs. Outpatient clinicians already spend a considerable amount of time engaging in shared decision-making about a variety of prevention- and treatment-related issues, especially in the primary care setting.^41^ Instead, other solutions are needed to help patients anticipate the financial burden of their medical care. In the clinic, pharmacists, social workers, or financial advisors could answer insurance coverage questions before or after visits and help patients apply for copay assistance. Insurance carriers could also make cost estimator tools available to patients online. These tools already exist for some insurance plans, but they have had very poor uptake,^40,42,43,44^ perhaps in part because patients who use them still have to contact their clinicians to request alternative medications or procedures when costs are too high. Better yet, insurance companies could simplify their cost-sharing mechanisms, especially as evidence grows that patients are bad at choosing insurance plans that provide the most coverage for the least amount of money.^12,45^ Finally, insurance companies and health systems could work together to make adjudicated, out-of-pocket costs available in the EHR at the point of prescription. In fact, the Centers for Medicare and Medicaid Services recently began requiring that all Medicare Part D plans adopt out-of-pocket cost estimators and make them compatible with at least one EHR.^46^ These tools have never been evaluated, however, so it is unknown whether they are accurate, what the optimal EHR design is, and how they affect prescribing patterns and patient outcomes.

Our study provides some hope that an EHR-based cost estimator could improve physician awareness of drug costs. Although we did not ask physicians to specify the details of their EHRs, those who reported having access to some information about out-of-pocket costs performed better than those who had no information. One possible explanation for this finding is that price transparency, however basic it may be, increases physicians’ awareness of costs and cost-sharing obligations. If that is the case, then point-of-care drug-related out-of-pocket cost information embedded in the EHR could increase the frequency and effectiveness of cost conversations, thereby improving patients’ decision-making capabilities.

### Limitations

This study had some limitations. It was limited by a response rate that was likely reduced because of COVID-19–related shutdowns in March 2020. Our results may therefore be affected by nonresponse bias. As the survey was anonymous, we could not evaluate for differences in characteristics between physicians who responded to the survey and physicians who did not respond. It is plausible that physicians who had difficulty answering our questions were less likely to respond to the survey, thus making our results an overestimate of physicians’ ability to accurately estimate out-of-pocket costs. Second, some physicians may have gotten the cost-sharing questions wrong because they did not take the time to do the math needed to answer the questions. If this did happen, it is likely a realistic illustration of the way these physicians approach cost conversations in the clinic. Third, we did not assess physician knowledge of available financial resources, such as pharmaceutical assistance programs and drug rebates. Future studies should evaluate the strategies that patients and clinicians use to alleviate out-of-pocket cost burdens, and how those strategies change depending on patient and clinician understanding of insurance cost-sharing mechanisms. Finally, this survey was targeted at internists and family medicine physicians, so our results are not generalizable to other specialties. Future research should explore understanding of insurance coverage and cost-sharing mechanisms among physicians in other specialties.

## Conclusions

This survey study found that most physicians agree with the importance of discussing costs with their patients but that few are able to accurately estimate out-of-pocket costs well enough to lead informed conversations about financial trade-offs with their patients. These findings suggest that increased price transparency and simpler insurance cost-sharing mechanisms are needed to enable informed cost conversations at the point of prescribing.

## Notes

Supplement. **eFigure.** Survey Sent to Primary Care Physicians **eTable 1.** Vignette Responses Defined to Be Correct in Sensitivity Analysis **eTable 2.** Rotated Factor Loadings for Physician Attitudes Toward Cost Conversations
