# Supplementary material for: Accuracy of Physician Estimates of Out-of-Pocket Costs for Medication Filling
Source: JAMA Netw Open. 2021 Nov 5;4(11):e2133188. doi: 10.1001/jamanetworkopen.2021.33188 (PMC8571653; doi:10.1001/jamanetworkopen.2021.33188)
Supplement: Supplement. — eFigure. Survey Sent to Primary Care Physicians eTable 1. Vignette Responses Defined to Be Correct in Sensitivity Analysis eTable 2. Rotated Factor Loadings for Physician Attitudes Toward Cost Conversations [file jamanetwopen-e2133188-s001.pdf]

Supplemental Online Content

Sloan CE, Millo L, Gutterman S, Ubel PA. Accuracy of physician estimates of out-of-pocket costs for medication filling. *JAMA Netw Open*. 2021;4(11):e2133188. doi:10.1001/jamanetworkopen.2021.33188

eFigure. Survey Sent to Primary Care Physicians

eTable 1. Vignette Responses Defined to Be Correct in Sensitivity Analysis

eTable 2. Rotated Factor Loadings for Physician Attitudes Toward Cost Conversations

This supplemental material has been provided by the authors to give readers additional information about their work.

© 2021 Sloan CE et al. *JAMA Network Open.*

eFigure. Survey sent to primary care physicians

Conversations About Cost in the Clinic: The Physician's Perspective

Dear Colleague:

In recent years, an increasing number of Americans have enrolled in health insurance plans that carry high out-of-pocket expenses. As a result, some patients experience a sizeable financial burden associated with their care. Currently, little is known about physicians' experiences discussing these costs with their patients.

With your answers to this anonymous survey, I hope to understand how physicians think about discussing out-of-pocket costs with their patients.

Before you take the survey, there are a few things you should know:

1. This survey is **anonymous**.
2. It should take you **no more than 8 minutes** to complete.
3. You can keep this $5 for completing the survey.
4. If you are unable to complete the survey, **please return this $5 using the enclosed envelope.**

Please fill out the questionnaire and return it in the self-addressed, stamped envelope. So we don't mail you another survey, please also mail back the enclosed stamped postcard separately from your survey.

Your response is critical. It is only with your personal help that our experiences as physicians can be better understood.

Thank you,

Caroline Sloan, MD
Department of Medicine
Duke University Health System
Durham VA Medical Center
caroline.sloan@duke.edu

© 2021 Sloan CE et al. *JAMA Network Open.*

First, please check the corresponding box to answer some questions about your beliefs and opinions. There are no right or wrong answers.

	Strongly Disagree	Disagree	Somewhat Disagree	Somewhat Agree	Agree	Strongly Agree
There is nothing I can do to lower patients' out-of-pocket costs.	☐	☐	☐	☐	☐	☐
Doctors have an obligation to initiate discussions about out-of-pocket costs when writing orders.	☐	☐	☐	☐	☐	☐
I know approximately how much my patients are spending on their medical care.	☐	☐	☐	☐	☐	☐
It is not my job to deal with patients' out-of-pocket costs.	☐	☐	☐	☐	☐	☐
My patients expect me to solve their cost-related issues during or right after clinic visits.	☐	☐	☐	☐	☐	☐
I have a hard time advising my patients on their out-of-pocket costs.	☐	☐	☐	☐	☐	☐

Please check the corresponding box to rate how much you agree or disagree that each of the following is a barrier that prevents you from discussing out-of-pocket costs.

	Strongly Disagree	Disagree	Somewhat Disagree	Somewhat Agree	Agree	Strongly Agree
I feel uncomfortable discussing out-of-pocket costs with my patients.	☐	☐	☐	☐	☐	☐
I do not have enough time to discuss patients' out-of-pocket costs.	☐	☐	☐	☐	☐	☐
I usually don't know how to answer questions about out-of-pocket costs.	☐	☐	☐	☐	☐	☐

© 2021 Sloan CE et al. *JAMA Network Open.*

Now, imagine the following:

- You are about to prescribe the oral PCSK 9 inhibitor evolocumab to your patient, Ms. Gray.
- One month's supply of evolocumab costs $1,000. She asks how much she'll have to pay out of pocket.
- Luckily, you have access to her private insurance information. Evolocumab is a tier 4 drug, and her insurance runs from January to December.

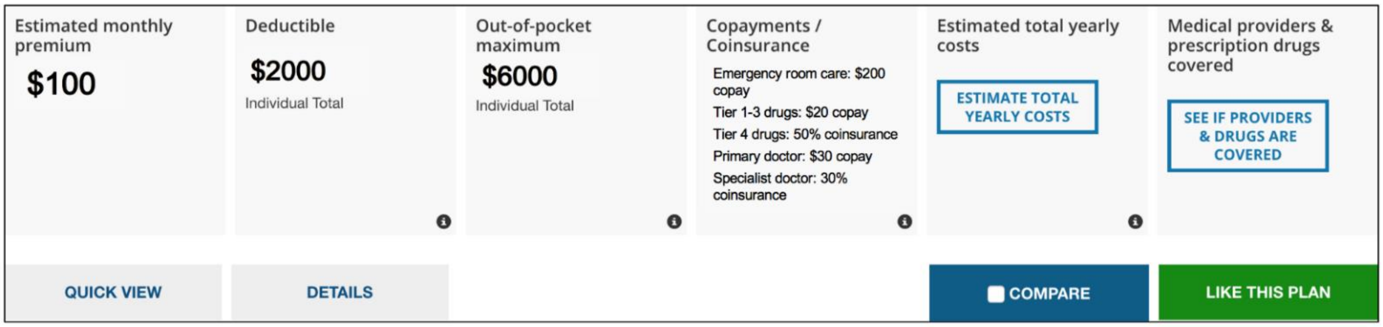

We know these questions are difficult to answer. Please provide your best guess of what her costs will be. Do not include premiums in your cost calculations.

1. It is January 3rd. How much will she pay out of pocket for just evolocumab this month?

   $____________________ in January ☐ I don't know

2. Now suppose she sees you again in March. Since January, she has had one clinic visit and taken two months' worth of evolocumab. What will she pay out of pocket for just the evolocumab this month?

   $____________________ in March ☐ I don't know

3. She also takes three other drugs, all tier 1. What will be the sum of her out-of-pocket costs for ***just those three drugs*** in March?

   $____________________ in March ☐ I don't know

4. Now suppose it's December. Since January, Ms. Gray has taken 11 months' worth of evolocumab. How much will she pay out of pocket for just evolocumab this month?

   $____________________ in December ☐ I don't know

© 2021 Sloan CE et al. *JAMA Network Open.*

Finally, please answer a few questions about yourself:

1. Gender:
   ☐ Male ☐ Female ☐ Do not wish to answer Other__________________

2. Are you of Hispanic, Latino/a, or Spanish origin?
   ☐ Yes ☐ No ☐ Do not wish to answer

3. Race (select all that apply):
   ☐ Black or African American ☐ Asian
   ☐ Native Hawaiian/Other Pacific Islander ☐ White
   ☐ American Indian/Alaska Native ☐ Do not wish to answer Other__________________

4. Year of birth: ______________________________

5. Year of medical school graduation: ______________________________

6. Approximate % of your time at work that is spent in:

   a) Outpatient clinical care: ____________________ b) Inpatient clinical care: ____________________
   c) Other (e.g. education, administration, research): ______________________________

7. Approximate % of patients in your practice who are covered by:

   a) Medicaid: ____________________ b) Uninsured: ____________________
   c) Private insurance or Medicare: ______________ d) Other: ____________________

8. Do you work in an academic medical center or teaching hospital?
   ☐ Yes ☐ No

9. Does your clinic have a social worker or financial counselor who can discuss financial issues with patients?
   ☐ Yes ☐ No ☐ Unsure

10. Do you use an electronic health record (EHR)?
    ☐ Yes ☐ No [IF NO, SKIP TO QUESTION 13]

11. Has your EHR enabled you to have more informed conversations about costs with your patients?
    ☐ Yes ☐ No ☐ Unsure

12. Does your EHR provide you with any information on out-of-pocket costs associated with your orders (e.g. copayments, retail prices, drug tiers)?
    ☐ Yes ☐ No ☐ Unsure

13. Is there anything else you would like to add about out-of-pocket costs and clinical practice?
    ____
    ____
    ____
    ____
    ____
    ____

14. If you would like to receive additional information about the study or answers to the insurance coverage questions, please provide your email address: ______________________________

© 2021 Sloan CE et al. *JAMA Network Open.*

eTable 1. Vignette responses defined as correct in sensitivity analysis

	Numerical responses	Correct vs Incorrect	Rationale for defining response as correct in the sensitivity analysis	Number of responses
Question 1. "It is January 3rd. How much will she pay out of pocket for just evolocumab this month?"				
	0	Incorrect	--	1
	5	Incorrect	--	1
	20	Incorrect	--	1
	50	Incorrect	--	2
	100	Incorrect	--	11
	250	Incorrect	--	1
	500	Incorrect	--	110
	600	Incorrect	--	2
	1000	Correct	Correct answer	192
	1030	Correct	+ primary care visit copay if deductible had been met	1
	1100	Correct	+ monthly premium	6
	1600	Incorrect	--	1
	2000	Incorrect	--	2
Question 2. "Now suppose she sees you again in March. Since January, she has had one clinic visit and taken two months' worth of evolocumab. What will she pay out of pocket for just the evolocumab this month?"				
	0	Incorrect	--	13
	5	Incorrect	--	1
	20	Incorrect	--	1
	50	Incorrect	--	2
	100	Incorrect	--	15
	200	Incorrect	--	1
	250	Incorrect	--	1
	300	Incorrect	--	2
	500	Correct	Correct answer	228
	530	Correct	+ primary care visit copay	1
	600	Correct	+ monthly premium	4
	1000	Incorrect	--	33
	1030	Incorrect	--	1
	1100	Incorrect	--	1
	1500	Incorrect	--	1
	2000	Incorrect	--	3
	2100	Incorrect	--	1
	2500	Incorrect	--	1
	3000	Incorrect	--	1
Question 3. "She also takes three other drugs, all tier 1. What will be the sum of her out-of-pocket costs for just those three drugs in March?"				
	0	Incorrect	--	6
	5	Incorrect	--	1
	12	Incorrect	--	1
	20	Correct	Included only one tier 1 drug (instead of 3)	1
	30	Incorrect	--	1
	60	Correct	Correct answer	224

© 2021 Sloan CE et al. *JAMA Network Open.*

	90	Correct	+ primary care visit copay	3
	100	Incorrect	--	3
	180	Incorrect	--	3
	300	Incorrect	--	3
	310	Incorrect	--	1
	380	Incorrect	--	1
	500	Incorrect	--	4
	520	Correct	Included only one tier 1 drug + the tier 4 drug	1
	540	Incorrect	--	4
	560	Correct	+ the tier 4 drug	32
	590	Correct	+ primary care visit copay + the tier 4 drug	1
	1000	Incorrect	--	3
	1060	Incorrect	--	3
	1130	Incorrect	--	1
	1500	Incorrect	--	1
	1560	Incorrect	--	1
	2060	Incorrect	--	1
Question 4. "Now suppose it's December. Since January, Ms. Gray has taken 11 months' worth of evolocumab. How much will she pay out of pocket for just evolocumab this month?"				
	0	Correct	Correct answer	210
	5	Incorrect	--	1
	50	Incorrect	--	2
	100	Correct	+ monthly premium	10
	200	Incorrect	--	1
	250	Incorrect	--	1
	340	Incorrect	--	1
	500	Incorrect	--	66
	600	Incorrect	--	2
	1000	Incorrect	--	5
	1100	Incorrect	--	1
	6000	Incorrect	--	2
	6500	Incorrect	--	1
	6720	Incorrect	--	1
Note: This table excludes “I don’t know” and blank responses to vignette questions.				

© 2021 Sloan CE et al. *JAMA Network Open.*

eTable 2. Rotated factor loadings for physician attitudes toward cost conversations

	Composite category		
	Duty	Perceived barriers	Perceived expectations
Doctors have an obligation to initiate discussions about out-of-pocket costs when writing orders	-0.5235		
There is nothing I can do to lower patients' out-of-pocket costs	0.3832		
It is not my job to deal with patients' out-of-pocket costs	0.5235		
I have a hard time advising my patients on their out-of-pocket costs		0.7662	
I do not have enough time to discuss patients' out-of-pocket costs		0.5509	
I usually don't know how to answer questions about out-of-pocket costs		0.7881	
I know approximately how much my patients are spending on their medical care		-0.431	
I feel uncomfortable discussing out-of-pocket costs with my patients		0.5134	
My patients expect me to solve their cost-related issues during or right after clinic visits			0.2542

© 2021 Sloan CE et al. *JAMA Network Open.*
